# Benefits of Camel Milk over Cow and Goat Milk for Infant and Adult Health in Fighting Chronic Diseases: A Review

**DOI:** 10.3390/nu16223848

**Published:** 2024-11-10

**Authors:** Razan S. Almasri, Alaa S. Bedir, Yazan K. Ranneh, Khaled A. El-Tarabily, Seham M. Al Raish

**Affiliations:** 1 Department of Nutrition, College of Medicine and Health Science, United Arab Emirates University, Al Ain 15551, United Arab Emirates; 201950110@uaeu.ac.ae (R.S.A.); 201950078@uaeu.ac.ae (A.S.B.); 2 Department of Pharmacy, College of Pharmacy, Al Ain University of Science and Technology, Al Ain 64141, United Arab Emirates; yazan.ranneh@aau.ac.ae; 3 Department of Biology, College of Science, United Arab Emirates University, Al Ain 15551, United Arab Emirates; ktarabily@uaeu.ac.ae

**Keywords:** antioxidant activity, anti-inflammatory properties, bioactive peptides, hypoglycemic effects, insulin-like proteins, lipid profile, metabolic health, therapeutic potential

## Abstract

The nutritional composition, antimicrobial properties, and health benefits of camel milk (CAM), cow milk (COM), and goat milk (GOM) have been extensively studied for their roles in managing diabetes and cardiovascular diseases (CVD). This review compares these milk types’ nutritional and therapeutic properties, emphasizing their applications in chronic disease management. CAM is rich in insulin-like proteins, vitamins, minerals, and bioactive compounds that benefit glycemic control and cardiovascular health. It also exhibits potent antioxidants, anti-inflammatory, and lipid-lowering effects, which are crucial for managing diabetes and reducing CVD risk factors. While COM and GOM provide essential nutrients, their impact on metabolic health differs. GOM is known for its digestibility and antihypertensive properties, whereas COM’s higher lactose content may be less suitable for diabetic patients. CAM’s unique nutritional profile offers distinct therapeutic benefits, particularly for diabetes and CVD management. Further research is needed to clarify its mechanisms of action and optimize its clinical application for chronic disease prevention and management.

## 1. Introduction

Cardiovascular disease (CVD) remains the primary cause of mortality in patients with type 1 and type 2 diabetes mellitus [1,2]. In addition to the inherent increase in mortality in diabetic subjects, when type 1 and type 2 diabetes mellitus is combined with manifestations of CVD, such as myocardial infarction or stroke, the mortality rate nearly doubles, leading to an estimated reduction in life expectancy of approximately 12 years [3]. CVDs are the leading cause of death globally, accounting for approximately 32% of all global deaths, with an estimated 17.9 million deaths in 2019, primarily due to heart attacks and strokes [4]. From 1990 to 2019, the prevalence of type 1 and type 2 diabetes mellitus in the world almost doubled, steadily increasing at a rate of 2.5% per year [5]. Since nutrition is a cornerstone in preventing chronic diseases, certain functional foods with a broad spectrum of biological properties are required [5].

Camel milk (CAM) is a crucial component of the Arabian Gulf region’s human diet, both in childhood and adulthood and is referred to as “white desert gold” [6,7]. Camels represent a valuable financial asset to several communities, particularly in arid zones, because they have adapted to harsh climates and provide milk, meat, and transportation [6]. Biologically, camels can produce 4–30 L of milk daily, even under severe environmental conditions, such as extreme temperatures, scarce pasture, and water shortages [7]. CAM’s lactation period lasts 9 to 11 months, with maximum lactation lasting 2 to 3 months. CAM has gained attention in the scientific community for its potential therapeutic properties for adults, especially in managing type 1 and type 2 diabetes mellitus and CVD [7]. Diabetes, a chronic metabolic disorder characterized by elevated blood glucose levels, can lead to chronic complications, including CVD, which remains a leading cause of morbidity and mortality among diabetic patients [8]. The unique composition of CAM, which includes insulin-like proteins, vitamins, minerals, and unsaturated acids, has been recommended to confer beneficial effects on glycemic control and cardiovascular health [9].

Interestingly, while most studies have focused on the hypoglycemic effects of CAM intake in diabetes management [10], scientific evidence suggests that CAM may have a role in mitigating cardiovascular risk factors [11]. The nutritional component of CAM, which differs significantly from that of cow milk (COM), includes elements that may exert antioxidant, antimicrobial, and ACE-inhibitory activities, potentially offering protection against CVD [12]. Nevertheless, the consumption of CAM has been associated with improved diabetes markers and may contribute to cardiovascular health. However, the mechanisms underlying these effects are not fully understood, and further research is still required to elucidate the precise bioactive constituents and their interactions with metabolic pathways [11,12]. In addition to these beneficial health effects of CAM in adulthood, it also provides beneficial nutrition in infancy and childhood [12].

The current review aims to explore the existing literature on the impact of CAM on diabetes and CVD, highlighting the potential benefits and the need for more comprehensive studies to confirm its efficacy dosage and understand the underlying mechanisms. This review compares CAM, COM, and goat milk (GOM), focusing on their nutritional content and therapeutic potential for disordered metabolic states and providing antimicrobial action. Prior research has investigated the advantages of different milk varieties separately; however, this review consolidates results from clinical trials, animal studies, and in vitro tests. This review also illuminates the bioactive components and their health effects, particularly in chronic disease prevention, to better understand these milk types’ of potential uses in modern healthcare. This comparative analysis provides crucial insights for future research, particularly in optimizing CAM clinical use for metabolic conditions.

## 2. Study Selection Criteria

We conducted a comprehensive literature search for this review using multiple databases, including PubMed, ScienceDirect, and Google Scholar. The following inclusion and exclusion criteria guided the selection process to ensure the relevance and quality of the studies.

### 2.1. Inclusion Criteria

- Studies published within the last six years (2018–2024) to reflect the most recent developments in the research on CAM, COM, and GOM.
- Peer-reviewed articles focused on the nutritional composition, hypoglycemic, lipid-lowering, antioxidant, or antimicrobial effects of CAM, COM, and GOM, particularly concerning diabetes, CVD, and other health conditions.
- Various study designs, including clinical trials, animal studies, and in vitro experiments, were considered to provide a comprehensive understanding of the potential health impacts of these milk types.
- Articles written in English are available in full-text format.

### 2.2. Exclusion Criteria

- Studies published before 2018 unless they provided critical data or insights foundational to the reviewed topics.
- Articles that did not specifically address the health effects or nutritional comparisons of CAM, COM, and GOM.
- Reviews and meta-analyses that did not offer new insights or perspectives beyond those found in more recent primary research.

While clinical studies were prioritized where available, relevant animal studies and in vitro research were included to support an understanding of underlying mechanisms. This inclusive approach allowed us to present a broad yet detailed assessment of the therapeutic potential of these milk types in managing diabetes, CVD, and antimicrobial activity.

## 3. Nutritional Composition of Camel Milk

Recent literature has revealed significant disparities in the composition of CAM [13,14,15,16]. Recently, meta-analysis studies that evaluated the gross composition of CAM, including one-humped and Bactrian varieties, reported an average fat content of 3.82 ± 1.08 g/100 mL, a total protein content of 3.35 ± 0.62 g/100 mL, a lactose content of 4.46 ± 1.03 g/100 mL, a total solids content of 12.47 ± 1.53 g/100 mL, and an ash content of 0.79 ± 0.09 g/100 mL [17]. Furthermore, the same study reported an average fat content of 4.14 ± 0.80 g/100 mL, a total protein content of 3.33 ± 0.52 g/100 mL, a lactose content of 12.69 ± 1.11 g/100 mL, a total solids content of 4.18 ± 0.72 g/100 mL, and an ash content of 0.76 ± 0.09 g/100 mL for milk from East African one-humped camels [17].

**Figure 1:**
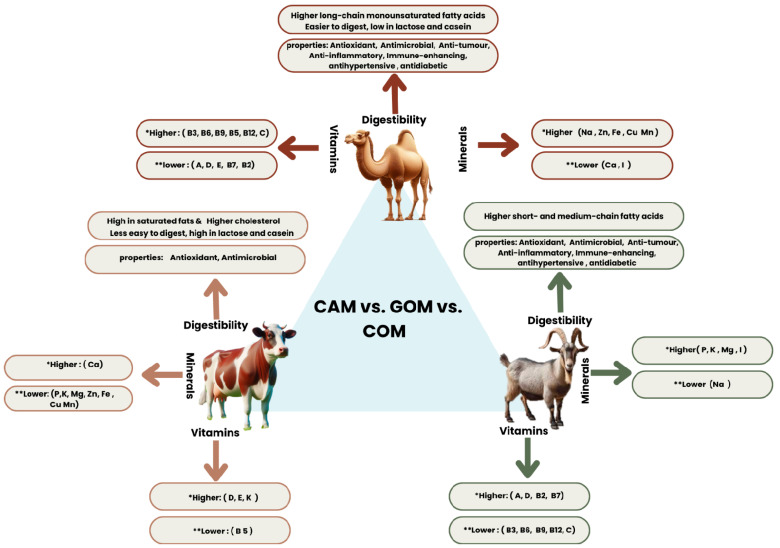
Comparison between camel, goat, and cow milk based on digestibility, minerals, and vitamins. CAM: camel milk; GOM: goat milk; and COM: cow milk. * Higher compared to the other two milk types, it has the highest content. ** Lower compared to the other two milk types, it has the lowest content. Ca, calcium; Fe, iron; P, phosphorus; K, potassium; Mg, magnesium; Cu, copper; Mn, manganese; Zn, zinc; Na, sodium; I, iodine; vitamin D, calciferol; vitamin A, retinol, or retinoic acid; vitamin B2, riboflavin; vitamin B3, niacin (or nicotinic acid); vitamin B12, cobalamin; vitamin B5, pantothenic acid; vitamin B9, folate (or folic acid); and vitamin B7, biotin [7,13,14,15,16].

The nutritional value of CAM is derived from its striking resemblance to human milk, which is comparable to mare and donkey milk [18]. CAM differs significantly from the milk of other ruminants, particularly in terms of its constituents [19,20,21] (Table 1). Unlike COM, CAM exhibits considerable component variation. One of the critical distinctions between ruminant milk and CAM is the absence of similarity in the physicochemical characteristics of their components. A comparison of CAM with GOM, bovine, and COM, in terms of composition, is presented in Figure 1. CAM has a higher ash content than other kinds of milk, whereas the lactose content in CAM is significantly lower than that in GOM, bovine, and COM (Figure 1) [19,21,22].

### 3.1. Protein

CAM consists of approximately 2.15% to 4.90% total protein, with an average value of 3.1 ± 0.5% [23,24]. Casein and whey protein are the main fractions of CAM protein. CAM contains approximately 1.63–2.76% casein, constituting the majority (52% to 87%) of the total protein [25]. The ratio of casein to whey protein in CAM is 73:27, and in GOM, it is 78:22 [26]. However, the ratio of casein to whey protein in COM is 79:20 [27]. GOM contains approximately 2.31% to 2.64% casein and 0.66% to 0.99% whey of total protein [24]. Compared to COM, CAM and GOM have higher concentrations of beta-casein, accounting for 65%-70% of the total casein content. In contrast, COM contains only 39% and 25–35% of total casein [24,28] consecutively.

In contrast, the kappa-casein percentage in GOM is 8.2%, and in CAM, it is 3.5% of the total protein, while COM has about 8–15% of the total casein content [24,27,28]. It is challenging for CAM to clot easily, so manufacturing fermented dairy products, such as yogurt and cheese, is still possible. The hurdle of producing CAM-fermented products is mainly associated with CAM proteins’ unique structural and functional properties. In particular, the low concentration of kappa-casein destroys the casein network during cutting and reduces the dry matter from cheese to whey [27,28].

The casein in CAM displays a distinct micellar size distribution compared to GOM casein, with an increased number of giant micelles present in CAM [29]. The diameter of most casein particles in GOM falls within the range of 180–220 nm, whereas CAM casein particles have a diameter of 350–380 nm [30,31]. However, the diameter of casein particles in COM is ranged from 100 to 200 nm [32]. The relatively low concentration of kappa-casein in CAM is associated with a large casein micelle and poor coagulation in response to acids or enzymes in contrast to GOM and COM [18,27]. The destabilization and coagulation of casein micelles during digestion and cheese production are mainly affected by the structural component of casein micelles. The site of chymosin cleavage in kappa-casein of CAM (comprising the amino acid Phe97-Ile98) is different from that present in GOM (comprising the amino acid Phe105-Met106). It is characterized by two additional proline residues at positions 95 and 105 [33].

CAM proteins comprise approximately 20–25% of whey proteins, representing the second most significant component of total proteins. These whey proteins, ranging from 0.62 to 0.81 g/100 g of milk, include a variety of immunoglobulins, such as lactoperoxidase, lactophorin, lactoferrin, peptidoglycan recognition proteins, lysozyme, serum albumin, and α-lactalbumin [34]. Although CAM contains a lower concentration of β-lactoglobulin than GOM, similar to COM, 77% of β-lactoglobulin in GOM is digested, while only 17% is digested in COM [33,34,35]. The denaturation of β-lactoglobulin and its association with kappa-casein at approximately 80 °C is crucial in forming firm yogurt gels from GOM [36]. On the other hand, the thin and fragile gel structure of yogurt made from CAM is due to the deficiency of CAM in β-lactoglobulin [33].

The influence of genetic factors on the compositional traits of CAM, COM, and GOM has been widely reported [37,38]. According to recent studies, beta-casein gene polymorphism has been linked to CAM, COM, and GOM [37,38] composition variations. The studies revealed that beta-casein gene polymorphism considerably impacted the acidity and protein content of Maghrebi CAM, Polish Holstein-Frisian COM, and Greek GOM [39,40]. In addition, three casein variants (α1-casein, β-casein, and κ-casein) have been found in Tunisian, Sudanese, and Nigerian camel breeds, leading to different milk compositions [41]. The genetic polymorphism of αs1-casein has been shown to affect milk lipid and protein composition, subsequently affecting the milk’s functional and nutritional properties [42]. Two genetic variants of αs1-casein have been identified in one-humped CAM, while four genetic variants of β-casein (A, B, C, and D) have been reported in GOM. Furthermore, three β-casein variants (A, B, and C) were fully characterized with 5P and 6P [40]. Also, two variants of β-casein (A1 and A2) were identified in Holstein-Frisian cows with a significant impact on producing large quantities of COM [39].

### 3.2. Lactose

The lactose content of CAM ranges between 35 and 49 g/L, which is slightly higher than that of COM [18]. GOM has a similar lactose concentration (approximately 39–41 g/L) [36]. During the lactation period, the lactose concentration in CAM remains relatively constant. Also, the influence of water intake after a drought has increased the lactose concentration from 2.8% to 3.8% within 24 h. However, the lactose concentration in GOM did not fluctuate significantly [36].

In contrast to COM, lactose content in GOM is elevated by dietary plant oil supplementation [36]. *Salsola*, *Atriplex*, and *Acacia* plants have been considered the favorable diet for a camel to achieve their salt requirements, resulting in CAM with a salty, sweet, or sometimes bitter taste [22]. Consumption of raw COM can pose a problem for lactose-intolerant individuals, as they cannot digest milk sugar lactose. While CAM contains a relative amount of lactose, compared to COM, lactose-intolerant individuals consume CAM without significant symptoms [12,22]. CAM is reportedly more digestible than COM in lactose-intolerant individuals [43]. The tolerance of CAM lactose could be due to the lower production of casomorphins, which subsequently lead to slower intestinal motility and affect lactose digestion [44].

### 3.3. Fat

The fat content in CAM can range between 2.9 and 5.4%, while GOM has a fat content of 3.4–4.2%. The individual globules of fat in GOM have a size of 2.76 μm, while the average fat globule size in CAM is 2.99 μm [22,45]. The small size of the fat globules in CAM and GOM contributes to their high fat digestibility but makes it challenging to produce butter, resulting in a lower yield. The fat content in CAM has higher concentrations of unsaturated fatty acids than other ruminant milk [46]. Compared with fat in COM, CAM contains higher concentrations of long-chain fatty acids [47]. CAM cream separates slowly and inadequately, with no skimmable cream forming after 48 h [22]. The lack of agglutinin protein in CAM is the primary cause of the reduction in cream formation [48].

Similarly, the creaming ability of GOM has also been attributed to the deficient amount of agglutinin and the smaller size of the fat globules [21]. CAM contains a lower proportion of short-chain fatty acids compared to COM fat. Nevertheless, long-chain monounsaturated fatty acids are more commonly found in CAM than in bovine, mare, or GOM [48]. In contrast, GOM has a higher concentration of short-chain and medium-chain fatty acids than COM and CAM [21]. GOM contains approximately 15–18% of its total fatty acid as short-chain fatty acids, higher than the 5–9% found in COM [21]. The odor of GOM is mainly attributed to the presence of short- and medium-chain fatty acids [21].

### 3.4. Minerals and Vitamins

The mineral and vitamin content of CAM in comparison to GOM is presented in Figure 1. CAM typically has lower calcium, phosphorus, potassium, and magnesium levels than GOM [49]. Specifically, CAM has calcium levels ranging from 114 to 116 mg/100 mL; COM contains 120 mg/100 mL, while GOM includes 134 mg/100 mL [49]. Similarly, phosphorus is present at 87.4 mg in CAM compared to 141 mg in GOM and 94 mg in COM, and potassium ranges from 144 to 156 mg in CAM against 181 mg in GOM and 142 mg in COM [49].

The magnesium content in CAM (10.5 to 12.3 mg) is also lower than in GOM (16 mg) and similar to COM (10–12 mg). However, CAM has a higher sodium content (59 mg) than GOM (41 mg) and COM (44 mg) [49]. In terms of trace elements, CAM tends to surpass GOM and COM. CAM has higher zinc levels (530 to 590 µg/100 g) than GOM and COM, with 560 µg/100 g and 400 µg/100 g, consecutively [49]. The intake of two cups from CAM (500 mL) can provide 70–90% of the recommended daily allowance for zinc and 192% of magnesium [49]. The higher zinc concentration in CAM is believed to be a significant factor in insulin secretion from beta-cell islets [7].

Iron content is considerably higher in CAM, ranging from 230 to 290 µg/100 g, than in GOM (70 µg/100 g), and COM (95 µg/100 g) [13]. CAM also contains more copper (140 µg) and manganese (80 µg) compared to both GOM, which has 50 µg of copper and 32 µg of manganese, and COM, which typically contains 30 µg of copper and 5 µg of manganese/100 g [14,15]. Notably, CAM does not detect iodine, whereas GOM contains 22 µg [13,15,16].

In terms of vitamins, CAM has lower levels of vitamins A, D, and riboflavin than in GOM and COM [13]. Specifically, CAM contains 26.7 µg of vitamin A and 0.3 µg of vitamin D. In comparison, GOM contains 55 µg of vitamin A and 0.69 µg of vitamin D, while COM contains 50 µg of vitamin A and 1 µg of vitamin D/100 g [13,15]. CAM also has less riboflavin, with 0.17 mg present compared to 0.21 mg in GOM. On the other hand, CAM is richer in niacin (0.77 mg), pyridoxine (0.55 mg), folic acid (87 µg), cobalamin (85 µg), and vitamin C (33 mg) compared to both COM and GOM, with COM containing 0.1 to 0.2 mg of niacin, 0.05 mg of pyridoxine, 5 to 10 µg of folic acid, 0.3 to 0.4 µg of cobalamin, and 0.5 to 2 mg of vitamin C [13,15,16]. In comparison, GOM contains 0.27 mg of niacin, 0.046 mg of pyridoxine, 1 µg of folic acid, 0.065 µg of cobalamin, and 1.29 mg of vitamin C. CAM also has slightly higher levels of pantothenic acid, with 0.37 mg compared to 0.31 mg in GOM and approximately 0.3 mg in COM [15,16]. However, biotin and vitamin B12 are not detected in CAM, while GOM contains 1.5 µg and 0.065 µg, and COM contains 2 to 3 µg of biotin and 0.3 to 0.4 of vitamin B12 [13,15,16]. One-quarter of a liter of CAM supplies approximately 10.5% of the recommended daily intake of vitamin C, vitamin B1, and vitamin B6, 8.25% of vitamin B2, 15.5% of vitamin B12, and 5.25% of vitamin A [13,15,16].

## 4. Milk Digestion

### 4.1. Infant Milk Digestion

Zou and his colleagues [50] have assessed the digestibility of human milk, CAM, and COM proteins using in vitro gastrointestinal digestion for infants. It was concluded that the digestion of CAM proteins in infants is affected by the higher gastric pH and lower levels of pepsin during the gastric phase [50]. During the early days of infant life, chymosin-like enzymes are produced to support milk protein digestion. However, the concentrations of these enzymes are reduced by the eleventh day [50]. Due to the higher pH levels in infant gastric, CAM forms casein, which forms a single clot that decreases the rate of gastric digestion and results in limited protein hydrolysis [50]. However, the in vitro model has demonstrated that CAM’s intestinal phase digestion is rapid and results in extensive protein hydrolysis [50].

Consequently, most studies on milk fat digestion have concentrated on the intestinal phase [51]. It is strongly advised that stomach lipases be included in the in vitro digestion tests because of their essential function in facilitating fat breakdown by intestinal lipases [51]. The significance of gastric lipases in infants is notable due to their increased postprandial gastric pH compared to adults [51].

Infants’ immature digestive system development produces lower stomach acid and enzyme concentrations [52]. COM’s infant digestion simulation system has revealed slower digestion of COM proteins. The formation of firm curds due to a high concentration of αs1-casein leads to slower protein hydrolysis than GOM [52]. Furthermore, the presence of β-lactoglobulin in whey protein fractions is more resistant to digestion and could cause allergic reactions in some infants [53]. The differences in protein structure and digestion kinetics of COM could affect nutrient absorption [53]. Several factors influence the digestion of COM fat in infants. The large globule size of COM fat (2.8 to 4.6 µm) requires additional effort by the infant digestion system, which is immature [53]. The high pH values in the infant’s stomach and lower concentration of pancreatic lipase delay the process of COM fat digestion. Furthermore, during digestion, fat globules in COM tend to destabilize and aggregate into larger particles, leading to additional resistance toward enzyme activity [53].

Studies have shown that the serum phase contains more proteins and solids during the digestion of GOM in infants than the cream phase [52]. This indicates that GOM proteins and fats remain more accessible to digestive enzymes, leading to faster gastric emptying and efficient digestion. Since GOM contains lower concentrations of αs1-casein than COM, softer curds occur, leading to efficient protein hydrolysis [52,53,54,55]. The elevated concentration of A2 β-casein in GOM alleviates stomach pain in infants [52,56]. Concerning fat digestion, GOM contains smaller fat globules, increasing the surface-to-volume ratio and enhancing the accessibility of pancreatic and gastric lipase, leading to the rapid release of free fatty acids [56].

### 4.2. Childhood Allergy to CAM

CAM allergies do not usually occur, despite the widespread rise in camels in nearly 44 countries and its inclusion as a central component of specific communities’ diets [57]. The medical literature has recently documented only one case of a child who experienced anaphylaxis as a result of consuming CAM [57]. IgE-mediated immune responses characterize this sort of allergy. A recent retrospective study found that allergy due to CAM is a distinct clinical condition characterized by rapid onset of skin urticaria, angioedema, and occasional anaphylaxis, which occurs within 15 min of ingestion [58]. The studied patients, with a strong familial predisposition, often have concurrent allergies, mainly atopic dermatitis, elevated eosinophil counts, and high IgE levels [58]. The skin-prick test is a reliable method for confirming a diagnosis of CAM allergy in individuals with a consistent history of related symptoms. In a study using sera from COM allergy patients, Restani et al. [59] did not find IgE binding with CAM’s proteins.

Nevertheless, El Agamy et al. [60] observed differences between COM’s protein and CAM’s epitopes. It was shown that children with allergic responses to COM were able to tolerate CAM without experiencing any side consequences. Most children with COM allergy could consume CAM safely without experiencing allergic reactions, as confirmed by negative skin-prick tests (SPT) [60]. These tests have proven reliable for identifying those who can tolerate CAM despite having an allergy to COM, providing a potential nutritional solution for managing the allergy [60].

### 4.3. Adult Milk Digestion

In adults, CAM proteins are digested primarily by the enzyme pepsin and gastric lipases in the stomach in hydrochloric acid. While pepsin effectively hydrolyzes the phenylalanine-methionine bonds of kappa-casein, the CAM proteins coagulate and form micelles [61]. Unlike COM, CAM and GOM form small and rapid evacuating particles of casein, by which the digestion is enhanced [61,62]. Pepsin demonstrates its maximum activity between pH 2 and pH 5, at which point protein hydrolysis is efficiently ensured. In light of this, understanding the coagulation behavior of CAM and the digestive dynamics during gastric digestion is of great significance because milk coagulation can influence the delivery levels of proteins, fats, and other nutritional milk compounds [63].

Previous investigations have examined CAM protein digestibility [64]. The simulation of gastrointestinal digestion using in vitro models has mirrored the digestive system of adults to observe closely the digestion of CAM [64]. Surprisingly, it was concluded that the gastric pH is significantly lower, and pepsin output is higher among adults [64]. In adults, about 10 to 20% of CAM fat is digested during the gastric phase via gastric lipases, while the rest of the fat is digested in the intestinal phase. The smaller sizes of CAM fat globules, ranging from 1.1 to 1.2 µm, play a significant role in enhancing fat digestion compared to larger fat globules found in buffalo (3.9–7.7 mm), COM (1.6–4.9 mm), and GOM (1.1–3.9 mm) [48]. Furthermore, these smaller fat globules provide a larger surface area for intestinal lipases to act in different locations and promote rapid lipid digestion [47].

CAM is particularly suitable for adults with lactose intolerance due to its lower concentration of casomorphin, which reduces intestinal motility and increases lactose exposure to lactase [65]. Additionally, the higher concentrations of L-lactate in CAM contribute to improving lactose digestion [18]. Research has been conducted to determine whether individuals with lactose intolerance could consume CAM without experiencing adverse events [66]. The results suggested that CAM could be a potential alternative for lactose-intolerant patients who experience symptoms after consuming COM [65,66].

COM digestion in adults is influenced by the composition, structure, and behavior of fat and proteins in the gastrointestinal tract. COM contains a higher proportion of αs1-casein, which forms firm curds in the stomach during digestion, leading to a delay in gastric emptying [63,65]. In addition, the aggregation of casein micelles in COM postpones the release of nutrients and protein breakdown due to the formation of dense clots under acidic conditions [15]. Furthermore, the fat globules of COM are approximately 2.8 to 4.6 µm in diameter. This size requires extra efforts by pancreatic lipases and bile salts to complete the process of emulsification and digestion [63].

GOM digestion in adults is generally easier than COM due to protein and fat composition. GOM forms smaller curds during digestion because of its lower concentration of αs1-casein. Using an in vitro digestion model, it was found that GOM contains fewer peptides of casomorphin-7 (BCM-7), which stimulate the intestinal epithelial cells to produce mucus [52]. The continuous stimulation of mucus production may affect the absorption and digestion processes [52]. The fat globule size of GOM ranges from 1.1 to 3.9 µm in diameter, which is smaller than the fat globule in COM [54]. Based on the in vitro digestion of GOM fat, the lipase enzyme has been more efficient in hydrolyzing fat molecules due to the smaller size of the fat globule, which increases the surface-to-volume ratio [54]. The levels of free fatty acids released from GOM fat globules occurred due to medium-chain fatty acids, which are higher than in CAM [54].

## 5. CAM and Metabolic Disorders in Adulthood

### 5.1. CAM and Diabetes

The utilization of CAM, COM, and GOM in managing chronic diseases like diabetes and CVD is profoundly affected by cultural practices and regional availability [66,67]. CAM, traditionally ingested in arid regions such as the Middle East and North Africa, has been a fundamental dietary component in these areas owing to the significance of camels in everyday life and sustenance [66,67,68,69]. The abundant presence of bioactive compounds, such as insulin-like peptides, antioxidants, and elevated zinc levels, renders it especially efficacious in glucose regulation and enhancement of cardiovascular health. Furthermore, CAM’s low lactose content and hypoallergenic characteristics make it more appropriate for lactose-intolerant populations, a prevalent characteristic in these areas [47]. Conversely, COM and GOM are more extensively consumed worldwide, with COM predominating in Western diets [67,68,69].

COM contains advantageous insulin; however, its elevated lactose content may restrict its consumption by lactose-intolerant individuals [70,71]. GOM, frequently ingested in rural and mountainous regions, has demonstrated the ability to enhance lipid profiles and reduce cholesterol, promoting cardiovascular health [72]. These milk varieties’ particular cultural preferences and regional availability influence their application and therapeutic efficacy in managing chronic diseases [71,72,73].

**Figure 2:**
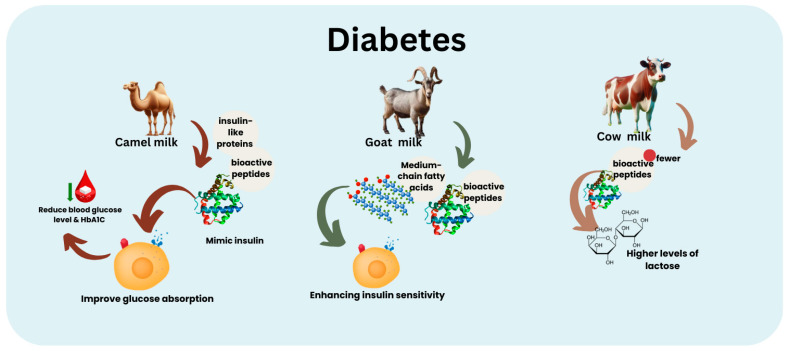
Comparison between camel, goat, and cow milk and their effects on diabetes. HbA1C: hemoglobin A1c test. Arrows indicate mechanisms: camel milk mimics insulin, improving glucose absorption; goat milk enhances insulin sensitivity; and cow milk has less bioactive peptides and a higher lactose content.

CAM is gaining recognition for its distinct composition and potential therapeutic advantages, especially in managing diabetes (Figure 2). CAM contains a higher concentration of insulin-like proteins and bioactive peptides compared to COM and GOM. These substances can imitate insulin and improve glucose absorption, reducing blood glucose levels [74]. Consistent intake of CAM has demonstrated the ability to lower blood sugar levels and HbA1C, thereby reducing the reliance on insulin in individuals with diabetes [75]. Compared to COM, CAM has elevated lactoferrin concentrations, immunoglobulins, lactoperoxidase, and lysozymes. These components are responsible for their anti-inflammatory, antioxidant, and immune-enhancing properties [76]. Including vitamin C, iron, and zinc in this dietary option enhances its nutritional value, making it a comprehensive choice for managing diabetes and other health conditions [77].

On the other hand, COM contains fewer bioactive compounds and higher levels of lactose, which may not be as advantageous for individuals with diabetes. Nevertheless, it continues to be an essential source of nutrients and is frequently enriched to enhance its nutritional composition [67]. GOM is recognized for being more accessible to digest and having less lactose. Additionally, it possesses anti-diabetic properties due to its bioactive peptides and medium-chain fatty acids. These components contribute to enhancing insulin sensitivity and offering antioxidant advantages [13].

Although COM is nutritionally dense, it does not contain the insulin-like proteins present in CAM. The elevated lactose content may concern lactose-intolerant individuals, including certain diabetic patients [64,77]. However, despite other options, COM is an essential component of many diets because of its widespread availability and rich nutritional composition [64].

CAM has been consumed worldwide for centuries because of its medicinal and curative attributes. Kenya and Somalia are prominent producers of goods, with Kenya’s annual production reaching approximately 1.165 million metric tons [65]. CAM is consumed in regions such as Uzbekistan in its raw form and as a fermented product, offering vital nutrients and promoting health advantages [25]. The composition of CAM can exhibit notable variations depending on factors such as breed, lactation stage, and feeding conditions. However, it consistently contains advantageous bioactive compounds that contribute to its therapeutic properties [78].

GOM contains a high concentration of bioactive compounds and has been proven to improve glucose and lipid metabolism in individuals with diabetes. It also benefits individuals who have lactose intolerance due to its smaller fat globules and increased digestibility [79]. GOM proteins have various health advantages, including immunomodulatory, anti-inflammatory, and antioxidant effects. They can treat autoimmune diseases and allergies [13]. Moreover, the consumption of GOM has been associated with improved hemoglobin levels and decreased rates of obesity, insulin resistance, and inflammation [79,80]. COM is abundant in vital nutrients and contains a more significant amount of carbohydrates than GOM. Its well-balanced combination of proteins, carbohydrates, and fats promotes a healthy gut [81]. Research has demonstrated that digesting milk fat from GOM and CAM is more effective than COM, indicating the superior ability of GOM and CAM fats to be digested [80,81].

### 5.2. Camel Milk and CVD

**Figure 3:**
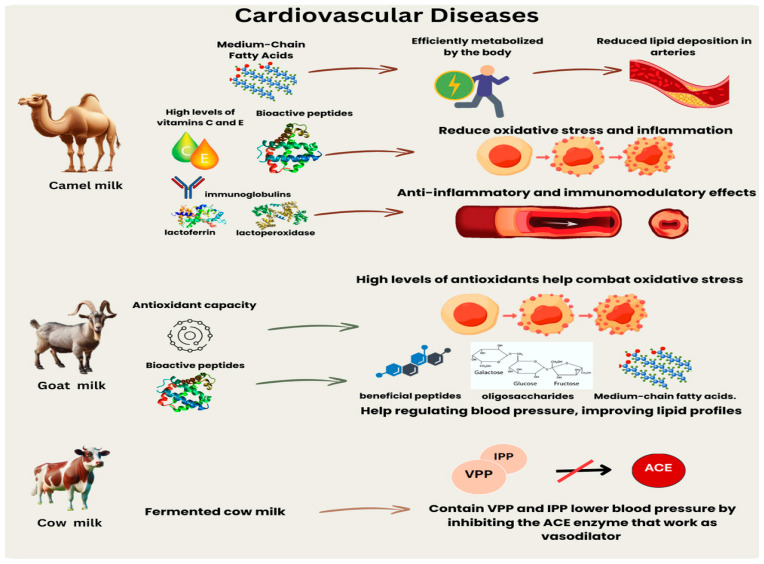
The relationship between camel, goat, and cow milk with cardiovascular diseases. Arrows indicate effects: camel milk reduces oxidative stress and inflammation; goat milk offers antioxidant support and aids in blood pressure regulation; and fermented cow milk decreases blood pressure by blocking the ACE enzyme. VPP, valyl prolyl proline; IPP, isoleucyl prolyl proline; ACE, angiotensin-converting enzyme.

The nutritional and therapeutic properties are gaining recognition, particularly their potential benefits in promoting cardiovascular health (Figure 3). CAM is rich in medium-chain fatty acids, which have been proven beneficial to cardiovascular health [81]. The human body can efficiently metabolize these fatty acids, using them as a readily accessible energy source [81,82]. This metabolic process reduces the likelihood of lipid deposition in the arteries [12].

CAM offers a significant cardiovascular benefit by effectively lowering cholesterol levels. Studies have shown that raw and fermented CAM significantly reduces triglycerides and cholesterol levels, essential to preventing CVD [81,82]. The ingestion of fermented CAM fortified with plant sterols resulted in a notable decrease in the atherogenic index and levels of small, dense LDL in rats with high cholesterol [82]. In addition, it led to a reduction in serum malondialdehyde (MDA) levels, which serves as an indicator of oxidative stress [82]. Furthermore, the potential benefits of CAM in diabetes management are closely linked to cardiovascular health. CAM helps to manage diabetes, a significant risk factor for CVD, by improving insulin sensitivity and reducing blood glucose levels. According to Sboui et al. [83], CAM contains insulin-like proteins that can help regulate blood sugar levels and reduce the need for external insulin in people with diabetes [83].

Milk from camels, cows, and goats has unique qualities and provides specific health benefits, particularly concerning CVD [83]. GOM is renowned for its capacity to prevent CVD due to its elevated antioxidant capacity and the presence of bioactive components such as peptides, oligosaccharides, and medium-chain fatty acids [73]. GOM contains anti-inflammatory, anti-diabetic, and anti-hypertensive compounds, contributing to its beneficial effects on cardiovascular health [13].

COM, extensively consumed worldwide, contains vital nutrients that support cardiovascular health. Research indicates that COM may possess a greater carbohydrate content than GOM, potentially influencing lipid metabolism in distinct ways [83]. Consumption of COM, owing to its elevated casein and calcium content, has been linked to heightened cholesterol levels [84]. Furthermore, although COM does not directly influence hemoglobin synthesis, its elevated calcium content may impede iron absorption, thereby indirectly affecting hemoglobin levels due to diminished iron bioavailability [83,84]. 

Compared to COM, CAM contains a higher concentration of medium-chain fatty acids, lower amounts of lactose, and is abundant in vitamin C and iron [85]. This substance has been found to have beneficial effects in managing CVD due to its anti-diabetic, anti-cancer, and antihypertensive properties. CAM contains a notable quantity of antioxidants, which can effectively reduce blood cholesterol levels and lipid peroxidation. This suggests that it can manage cardiovascular health [8,12].

Like COM, it is a prominent worldwide provider of sustenance, but it displays significant disparities in fat and protein composition [85]. Studies suggest that the decomposition of COM results in different lipid compositions, which may have diverse impacts on cardiovascular health compared to the milk of GOM and CAM. COM harbors bioactive peptides that benefit cardiovascular well-being [50,85].

## 6. CAM Antioxidant, Anti-Inflammatory, and Antimicrobial Properties

CAM has powerful antioxidant properties that are crucial for preserving cardiovascular health. The high levels of vitamins C and E and bioactive peptides contribute significantly to its ability to reduce oxidative stress and inflammation, both of which are linked to CVD [85]. Antioxidants assist in the removal of detrimental free radicals, thereby protecting the body from oxidative damage [84].

Furthermore, CAM is rich in bioactive proteins like lactoferrin, immunoglobulins, and lactoperoxidase, which have anti-inflammatory and immunomodulatory effects [85]. These properties have the added benefit of enhancing cardiovascular health by reducing inflammation and improving immune responses, ultimately decreasing the risk of heart disease [20]. Moreover, research has demonstrated that CAM can enhance the breakdown of fats and stimulate the production of enzymes that act as antioxidants. In a study involving rats fed a high-cholesterol diet, the introduction of CAM led to a significant improvement in lipid profiles [85]. This resulted in a reduction in total cholesterol, LDL cholesterol, and triglyceride levels, along with an elevation in HDL cholesterol levels. Khalid et al. [85] suggested that CAM can enhance cardiovascular well-being by facilitating a balanced lipid profile.

The antimicrobial properties of CAM, COM, and GOM are distinct because of their unique compositions of bioactive compounds. CAM contains abundant antimicrobial substances, including lactoferrin, lysozymes, and immunoglobulins [86]. These components enhance its effectiveness against harmful microorganisms and contribute to its extended storage duration, surpassing COM’s [86]. Moreover, CAM is a prominent diet component in numerous dry and semi-dry areas. It is renowned for its abundant health-enhancing compounds such as bioactive peptides, zinc, and mono- and polyunsaturated fatty acids [86]. These substances contribute to its ability to combat microorganisms, regulate blood sugar levels, protect against oxidative stress, and reduce cholesterol levels. The antimicrobial properties of CAM can be attributed to various components, including lactoferrin, lysozymes, lactoperoxidase, hydrogen peroxide, and immunoglobulins [86,87]. These components have proven effective against bacteria, including *Staphylococcus aureus*, *Listeria monocytogenes*, and *Escherichia coli.* Furthermore, including these antimicrobial agents enables CAM to withstand contamination and prolong its storage duration [77].

CAM is known for its anti-diabetic properties, primarily because it contains insulin-like proteins and immunoglobulins affecting the pancreas’ function and secretion. This can be beneficial for managing diabetes [87]. Furthermore, due to its inherent bioactive constituents, CAM has historically been employed for treating ailments such as tuberculosis, asthma, and jaundice [77]. CAM’s distinctive composition, which contains higher concentrations of vitamin C compared to COM, renders it especially valuable in areas where access to fresh produce is restricted. The results emphasized the diverse advantages of CAM, showcasing its potential as both a dietary supplement and a therapeutic remedy for various health conditions [47].

GOM has elevated concentrations of specific amino acids and demonstrates exceptional functional characteristics, such as antimicrobial properties. This makes it capable of combating detrimental bacteria and suitable for creating novel food items [13]. COM exhibits antimicrobial properties, especially when fermented with lactic acid bacteria. This fermentation process enhances its antioxidant activity and sensory characteristics [88]. These variations highlight the significance of comprehending the distinct constituents and advantages of various milk types to maximize their utilization in terms of health and nutrition [88].

GOM and CAM stand out for their high concentrations of bioactive compounds and exceptional antioxidant properties compared to other milk varieties. These characteristics make them particularly efficient in preventing and treating CVD, offering clear advantages for heart health [88]. CAM’s distinctive composition, rich in medium-chain fatty acids, antioxidants, bioactive proteins, and low lactose levels, makes it a valuable dietary component for preventing and managing CVD. This functional food’s cardiovascular health benefits are demonstrated by its capacity to improve lipid profiles, reduce oxidative stress and inflammation, and effectively control diabetes [88].

To summarize, GOM and CAM have higher levels of bioactive compounds and superior antioxidant properties than other milk types. These qualities make them particularly effective in preventing and managing CVD, offering unique cardiovascular benefits [88,89].

## 7. CAM and Gut Microbiota

The distinctive compositions and bioactive constituents of CAM, COM, and GOM each contribute uniquely to the modulation of gut microbiota [86]. CAM regulates the immune system and has anti-inflammatory, anti-apoptotic, and anti-diabetic properties [90]. These properties are due to the high levels of β-casein and various antibodies found in CAM. These properties exhibit enhanced antibacterial and antiviral efficacy compared to COM [86]. In addition, CAM improves the composition of gut microbiota by promoting the growth of beneficial bacteria such as *Bacteroides* and reducing the presence of harmful bacteria like *Dubosiella* in conditions such as non-alcoholic fatty liver disease [89]. The consumption of COM is prevalent, and it promotes the health of the digestive system due to its varied microbial composition and rich nutritional content. This significantly advances social and economic development [81].

Overall, while all types of milk have unique advantages in influencing gut microbiota, CAM and GOM stand out due to their superior digestibility and specific health-promoting properties. This information educates the audience about the potential benefits of this milk, enhancing their understanding of dietary interventions and therapeutic remedies [88,89].

## 8. Molecular Mechanisms in Beneficial Health Effects of CAM, COM, and GOM

CAM, COM, and GOM’s hypoglycemic, antihypertensive, and lipid-lowering properties are attributed to unique biochemical mechanisms, each enhancing metabolic health [90]. CAM demonstrates hypoglycemic effects via insulin-like proteins that facilitate glucose absorption into cells and improve β-cell functionality in the pancreas [90,91]. Moreover, CAM diminishes insulin resistance by decreasing inflammatory markers, including TNF-α and IL-6 [84]. The antihypertensive effects are mainly attributed to bioactive peptides that inhibit angiotensin-converting enzymes (ACEs), thereby reducing the synthesis of angiotensin II, a potent vasoconstrictor [73,85,92]. The elevated potassium levels in CAM contribute to blood pressure regulation by facilitating vasodilation [84,93,94]. CAM contains lactoferrin, which diminishes cholesterol synthesis by inhibiting the HMG-CoA reductase enzyme, while omega-3 and omega-6 fatty acids enhance the lipid profile by reducing LDL cholesterol and elevating HDL cholesterol [48,84,85,95,96,97,98].

COM exhibits hypoglycemic properties primarily due to its whey proteins, which promote the secretion of insulinotropic hormones like GLP-1 and GIP, thereby augmenting insulin release [99,100]. The branched-chain amino acids additionally stimulate insulin secretion [99,100,101,102]. The antihypertensive properties of COM arise from casein-derived peptides that inhibit ACE [103], akin to CAM, while its elevated calcium content promotes vascular relaxation. The lipid-lowering properties of COM are attributed to conjugated linoleic acid (CLA), which diminishes adipose tissue formation and augments lipid oxidation in conjunction with phospholipids that facilitate cholesterol excretion by elevating bile acid synthesis [104,105,106].

The hypoglycemic effects of GOM are attributed to its high concentration of medium-chain fatty acids (MCFAs), which are swiftly metabolized, enhancing insulin sensitivity and glucose utilization [107]. The oligosaccharides regulate the gut microbiota, diminishing inflammation and improving metabolic health [107]. The antihypertensive properties of GOM are ascribed to bioactive peptides with ACE inhibitory effects and its elevated magnesium content, which facilitates vasodilation. GOM’s medium-chain fatty acids enhance fat oxidation, while its prebiotic constituents foster a healthy gut microbiome, thereby optimizing lipid profiles [108,109,110]. The varied biochemical pathways highlight the potential of CAM, COM, and GOM’s potential as functional foods for managing diabetes, hypertension, and dyslipidemia [107,108,109,110].

## 9. Comparative Analysis of Human Milk, CAM, GOM, and COM: Nutritional Composition and Digestibility

Human milk, CAM, GOM, and COM have unique similarities and differences in their composition that impact their nutritional value and suitability for specific dietary needs [110]. Human milk stands out due to its low protein, fat, and mineral content but is rich in lactose, providing optimal nutrition for infant development [90,91]. It is also characterized by a high concentration of oligosaccharides, which are critical in promoting the growth of beneficial gut bacteria and supporting immune functions [111,112].

On the other hand, CAM is similar to human milk in some respects, such as its lower saturated fat content and lack of β-lactoglobulin, an allergen found in COM [50,113]. CAM is rich in minerals like iron and zinc. It contains higher levels of monounsaturated fats compared to human milk [113], making it a potential alternative for individuals with dairy sensitivities [114].

GOM has a higher content of medium-chain fatty acids, which are easier to digest than COM. It also contains less lactose, making it a better choice for lactose-intolerant people [112,115]. Though lower than human milk, GOM’s oligosaccharide content still contributes to prebiotic functions [112]. COM is more widely consumed but contains higher levels of saturated fats and proteins that can be harder to digest, particularly for infants. It forms a firmer curd in the stomach, which can delay digestion compared to the softer curd formed by humans or CAM [50]. COM also contains higher levels of casein, which may contribute to allergies in some individuals [111].

Human milk is ideally suited for infants, offering a balance of nutrients and immune-supporting components [116]. CAM and GOM provide some nutritional benefits for those with lactose intolerance or allergies to COM. In contrast, COM remains a common dietary staple but may not be as easily digestible or allergen-free as the other types [117]. Human milk has a unique nutritional profile due to physiological factors [116]. The mother’s nutritional, hydration and metabolic health significantly affect milk quality and quantity [117]. Lactogenesis and milk ejection depend on prolactin and oxytocin levels [116,117].

Maternal nutrition also affects breastmilk micronutrients like fat-soluble vitamins and essential fatty acids [118]. Milk composition is also affected by environmental stressors, maternal mental health, and sleep and exercise [118,119]. Comparing human milk to CAM requires an understanding of these physiological factors. Although CAM has similar bioactive constituents and saturated fat levels to human milk, it is less susceptible to physiological and environmental factors due to the absence of β-lactoglobulin [119]. CAM can satisfy nutritional sensitivities and dietary restrictions [50,113,120].

## 10. Conclusions

This review has practical implications for healthcare providers and nutritionists beyond its nutritional and therapeutic benefits. Patients with chronic conditions like diabetes and CVD can benefit from drinking CAM, COM, and GOM. Evidence-based recommendations for each milk type are below:

CAM: With its insulin-like proteins, antioxidants, and anti-inflammatory properties, CAM can help manage diabetes. It lowers blood glucose and improves insulin sensitivity, making it a good choice for diabetics. Because of its low lactose content, CAM suits lactose-intolerant people. For diabetics and CVD patients, the milk’s vitamin C, iron, and bioactive peptides improve cardiovascular health. Healthcare providers may recommend CAM as part of a balanced diet to control glycemic levels and reduce cardiovascular risk.

GOM: It is digestible and contains bioactive antihypertensive and antioxidant components. Its lower lactose content makes it more palatable for mild lactose intolerance. GOM may improve hypertensive and CVD patients’ lipid profiles and blood pressure. Its bioactive peptides improve insulin sensitivity, making it a valuable ingredient in cardiovascular and metabolic diets.

COM: Due to its high lactose content, diabetics, especially those with lactose intolerance, may not use COM. However, due to its high calcium and protein content, fortified COM can help manage cardiovascular health for patients who tolerate it. Adding COM to a balanced diet may improve cardiovascular function, especially in dairy-consuming populations.

In conclusion, the current review article emphasizes the promising prospects of CAM as a therapeutic agent for managing diabetes and CVD. The nutritional composition of CAM, which includes insulin-like proteins, vitamins, minerals, and bioactive compounds, provides notable health advantages. Scientific evidence indicates that regularly consuming CAM can enhance the control of blood sugar levels, decrease risk factors related to heart health, and improve overall metabolic well-being. Although these promising discoveries have been made, the exact mechanisms through which CAM produces advantageous effects are still uncertain. Further comprehensive and well-designed studies are necessary to fully elucidate the bioactive constituents of CAM and their interactions with metabolic pathways. This knowledge will be crucial in validating the therapeutic potential of CAM and optimizing its use in clinical settings for the prevention and management of chronic diseases.

Future research should focus on clinical trials that investigate specific bioactive compounds in CAM, such as insulin-like proteins and lactoferrin, to elucidate their mechanisms of action. These trials should evaluate the efficacy of CAM across various populations, specifically individuals with type 2 diabetes, cardiovascular conditions, lactose intolerance, or milk allergies. Further research is required to ascertain CAM consumption’s ideal dosage and long-term impacts across diverse age demographics and health statuses. Particular attention must be directed toward the interactions between bioactive compounds in CAM and metabolic pathways, which may provide insights into its potential for managing additional chronic conditions, including hypertension and dyslipidemia. These recommendations seek to direct future research and enhance the clinical utilization of CAM for disease prevention and management.

## Figures and Tables

**Table 1:** The nutritional and protein composition of camel, cow, and goat milk.

Component	Camel Milk	Cow Milk	Goat Milk	Reference
Fat (%)	3.82–5.4	3.0–4.0	3.4–4.2	[17,18]
Protein (%)	2.15–4.90	3.2–3.4	3.1–3.8	[17,18]
Casein (%)	1.63–2.76	2.5–2.7	2.31–2.64	[23,24]
Whey Protein (%)	0.6–0.7	0.6–0.7	0.66–0.99	[23,24]
Ash (%)	0.79–0.81	0.75	0.82	[17,18]
Calcium (mg/100 mL)	114–116	120–130	134	[17,18]
Vitamin C (mg/100 mL)	33	2	1.29	[17,18]
